# Effect of Activin Type II Receptor Signaling in Patients With HFpEF and Pulmonary Hypertension: A Critical Analysis of Biological Mechanisms and Clinical Trials

**DOI:** 10.1161/CIRCULATIONAHA.126.081957

**Published:** 2026-10-27

**Authors:** Milton Packer, Mardi Gomberg-Maitland, Ryan J. Tedford, David Langleben, Jean-Luc Vachiery, Barry A. Borlaug

**Affiliations:** 1 Baylor Heart and Vascular Institute, Dallas, TX and Imperial College, London, UK (M.P.).; 2 Department of Medicine, Section of Cardiology, George Washington University School of Medicine and Health Sciences, Washington, DC (M.G.-M.).; 3 Division of Cardiology, Department of Medicine, Medical University of South Carolina 2345 , Charleston, SC (R.J.T.).; 4 Department of Medicine, Section of Cardiology, Center for Pulmonary Vascular Disease, Azrieli Heart Center, Jewish General Hospital, McGill University, Montreal, Canada (D.L.).; 5 Department of Cardiology, Hôpital Universitaire de Bruxelles Erasme, Brussels, Belgium (J.-L.V.).; 6 Department of Cardiovascular Medicine, Mayo Clinic, Rochester, MN (B.A.B.).

**Keywords:** activin A, adipokine, heart failure with preserved ejection fraction, sotatercept

## Abstract

The totality of evidence indicates that the mechanisms underlying the increase in pulmonary vascular resistance in patients with combined postcapillary and precapillary pulmonary hypertension (CpcPH) associated with heart failure with preserved ejection fraction (HFpEF) differ meaningfully from those responsible for the increase in pulmonary vascular resistance in patients with pulmonary arterial hypertension. CpcPH-HFpEF is a complex disorder involving the pulmonary veins, left atrium, and pulmonary arterioles, but recent evidence has highlighted a potentially central role for pulmonary venous and left atrial (LA) dysfunction. Epicardial adipose tissue occupies anatomical proximity to both structures, allowing derivative secretory products to influence their structure and function. The most well-characterized proinflammatory adipokine released from an expanded epicardial fat mass is activin A, which causes fibrosis and cardiomyocyte stiffness and senescence in underlying tissues by signaling through activin type II receptor signal transduction. Such signaling regulates pulmonary venular responses to mechanical stretch, and circulating activin A is elevated in patients with HFpEF who have enlarged LA chambers, pulmonary venous hypertension and increased pulmonary vascular resistance. The adipokine hypothesis of HFpEF identified activin A and activin type II receptor signaling as key adipokine mediators in the pathogenesis of HFpEF. Experimentally, activin A signaling promotes the development of both atrial myopathy and HFpEF, and activin type II receptor ligand traps alleviate HFpEF and ameliorate both the pulmonary vascular and left heart abnormalities in experimental models in which CpcPH-HFpEF is induced by obesity. These collective observations led to the prospective hypothesis that adipokine modulation by sotatercept would mediate benefits on the left heart in patients with CpcPH-HFpEF. These expectations were validated when the CADENCE trial showed that sotatercept decreased both pulmonary artery wedge pressures and left atrial volume index in patients with CpcPH-HFpEF, which are effects that could not explained by a decrease in pulmonary vascular resistance. Activin A released from an expanded epicardial adipose tissue promotes both pulmonary venous and left atrial remodeling and fibrosis in vivo, which (in turn) are effects that can be attenuated by an activin A decoy. The efficacy of sotatercept to slow the progression of CpcPH-HFpEF is now being tested in a phase III outcomes trial.

Sotatercept is an activin type II receptor (ActRII) ligand trap, which binds to and sequesters the endogenous receptor ligands, particularly activin A.^1^ Activin A exerts proinflammatory and profibrotic vascular effects, and is overexpressed in remodeled pulmonary arterioles from patients with clinical pulmonary arterial hypertension (PAH).^2,3^ Sotatercept ameliorates the pulmonary vasculoproliferative and angio-obliterative lesions of experimental PAH,^4^ and it improves the clinical status and mitigates disease progression in patients with PAH.^5,6^

## Drugs for the Treatment of PAH: Distinguishing Pulmonary Vasodilators From Pulmonary Vascular Anti-Remodeling Drugs

Most drugs approved for PAH are considered to have direct or indirect pulmonary vasodilator properties. The pulmonary arterioles show increased expression of endothelin-1 and suppressed prostacyclin synthase expression and impaired nitric oxide–soluble guanylyl cyclase signaling. In PAH, prostacyclin and derivative analogs (eg, epoprostenol, treprostinil, iloprost, and selexipag), soluble guanylate cyclase stimulators (eg, riociguat), phosphodiesterase type-5 inhibitors (eg, sildenafil citrate and tadalafil), and endothelin receptor antagonists (eg, bosentan, ambrisentan, and macitentan) decrease pulmonary vascular resistance (PVR) by ≈15% to 30%, while reducing the risk of worsening clinical events by ≈25% to 50%.^7–11^ In contrast, the mechanism of action of sotatercept involves antiproliferative and antifibrotic effects that specifically target pulmonary vascular remodeling rather than pulmonary arteriolar tone.^12^ No drug decreases pulmonary artery wedge pressures (PAWP) or left atrial (LA) volumes in PAH.^5–10^

## Mechanism of Increased PVR in Combined Postcapillary and Precapillary Pulmonary Hypertension: A Disease of the Epicardial Adipose Tissue-Pulmonary Vein-Left Atrium Juncture?

Combined postcapillary and precapillary pulmonary hypertension (CpcPH) is characterized by increased PA pressures, PAWP and PVR, which are related to left-sided heart disease causing chronic pulmonary venous hypertension. In most patients, the disorder that afflicts the left heart is heart failure with preserved ejection fraction (HFpEF), and in this discussion, these patients are designated as having CpcPH-HFpEF.

### Is the Increased PVR in CpcPH-HFpEF Related to Pulmonary Arteriolar Vasoconstriction?

It is commonly believed that the mechanisms that elevate PVR in CpcPH-HFpEF are similar to those in PAH. Long-standing pulmonary venous hypertension can lead to medial hypertrophy, intimal proliferation, and adventitial thickening of the PA; however, in contrast to PAH, plexiform lesions, in situ thrombosis of pulmonary arterioles, and obliterative vasculopathy are typically absent in CpcPH. More importantly, drugs that are hemodynamically and clinically effective pulmonary vasodilators in PAH do not generally decrease PVR or lead to clinical benefits in CpcPH-HFpEF. In numerous randomized, placebo-controlled clinical trials of CpcPH-HFpEF, conventional pulmonary vasodilators have produced no or only modest changes in PVR after 12 to 24 weeks of double-blind therapy.^13,14^ Furthermore, treatment with pulmonary vasodilators has not improved symptoms, circulating natriuretic peptide concentration or exercise capacity. The effects of pulmonary vasodilators in PAH and in CpcPH-HFpEF trials are strikingly different.

Interestingly, if pulmonary vasodilators were to yield a meaningful decrease in PVR, such an effect might be undesirable in patients in whom left-sided pressures are already elevated. Pulmonary vasoconstriction and vascular remodeling have been regarded as mechanisms that protect the pulmonary capillaries from volume overload. Drug-induced decreases in PVR might increase transpulmonary blood flow and can lead to increased PAWP and lung congestion in patients with left-sided heart disease, accompanied by an increased risk of adverse clinical events.^13,14^

### Is the Increased PVR in CpcPH-HFpEF Related to Structural Changes in the Left Atrium and Pulmonary Veins?

Although studies of HFpEF have traditionally focused on left ventricular (LV) diastolic filling, patients with CpcPH-HFpEF typically exhibit important changes in LA structure and function indicative of an LA myopathy (eg, increases in LA volume and stiffness and decreases in LA strain).^15–17^ The severity of LA abnormalities is associated with worse symptoms and exercise tolerance and an increased risk of adverse heart failure outcomes. Yet, patients with CpcPH-HFpEF do not differ from patients with HFpEF without increased PVR with respect to LV wall thickness or diastolic filling abnormalities.^18^ These findings suggest that LA myopathy is an under-recognized feature of CpcPH-HFpEF.

Importantly, there is compelling evidence that the structural and pathophysiological changes in the left atrium in CpcPH-HFpEF extend to involve the pulmonary veins (PVs). The mechanism that is responsible for the increase in PVR seen in patients with left heart disease appears to be related to pulmonary venular (at least as much, if not more, than arteriolar) remodeling,^19^ and patients with CpcPH-HFpEF exhibit more (and not less) lung congestion at the same exertional LA pressure, suggesting a significant role for pulmonary venous disease in HFpEF.^19^ Accordingly, a significant anatomical site that drives the increased PVR observed in CpcPH-HFpEF resides at the level of the PVs and pulmonary venules, rather than the arteries and arterioles.

It is noteworthy that the LA main body is a thin-walled non-compacted structure that arises embryologically as an outgrowth of the PVs; the 2 present as histologically identical structures of vessel wall covered by extrapericardial-differentiated myocardium.^20,21^ Like the PVs, the LA main body serves a conduit function, being inherently stiff and with little intrinsic endocrine function. In contrast, the LA appendage is derived from the embryonic heart tube, and it consists of rough trabeculated endocardial and myocardial layers without a vessel wall component. The LA appendage is capacious and contractile, and synthesizes natriuretic peptides, but it is 6- to 8-fold smaller than the LA main body.

### Influence of Epicardial Adipose Tissue on the Structure and Function of PV–Left Atrium Juncture

Epicardial adipose tissue overlies the juncture of the PVs and LA main body.^22^ This fat depot originates from the splanchnopleuric mesoderm, which also gives rise to mesenteric and omental fat, whereas pericardiac fat is derived from the primitive thoracic mesenchyme. Epicardial fat is most prominent around the right ventricle and left atrium, and it wraps around the PVs as they enter the left atrium.^22^ Importantly, the epicardial adipose depot shares an unobstructed circulation with the underlying tissues, allowing its secretions to be transmitted through paracrine and vasocrine effects. Therefore, epicardial adipose tissue is well-positioned as a source of molecular signals that influence both the PVs and the left atrium (Figure 1). The expansion of epicardial fat may explain why obesity and visceral adiposity are associated with increased pulmonary venous pressures and pulmonary venous resistance in patients without HFpEF and with decreased pulmonary vasodilator reserve in patients with HFpEF.^23–25^

**Figure 1.**
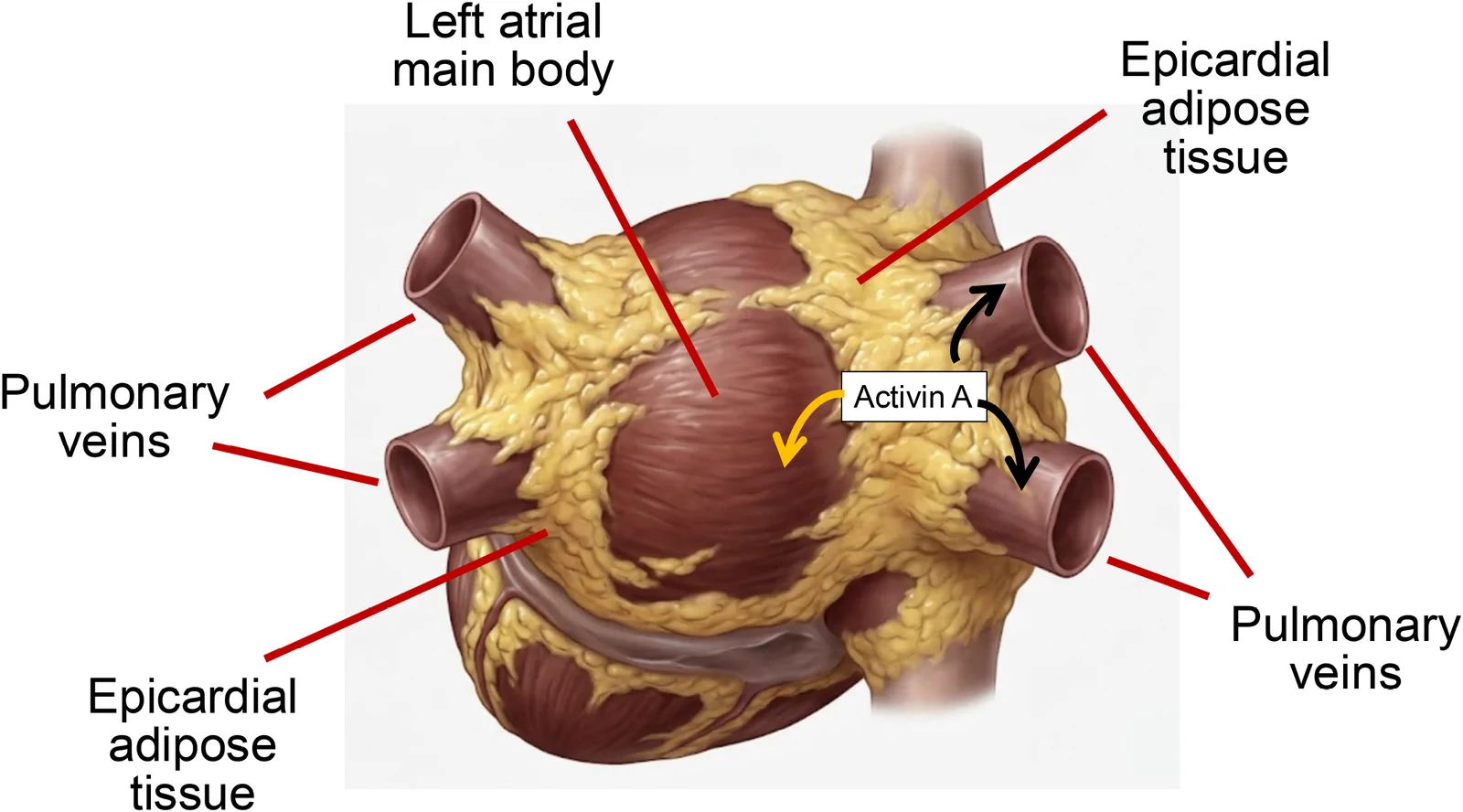
**Proximity of epicardial adipose tissue to the left atrial main body and pulmonary veins allows paracrine and vasocrine secretion of activin A.** The left atrial main body and the pulmonary veins share embryological origins and histological features, and epicardial fat overlays both structures. In states of excess visceral adiposity, a prominent signaling molecule secreted by an expanded epicardial adipose tissue mass is activin A, which promotes inflammation and fibrosis onto the adjacent structures, with which it shares an unobstructed circulation.

In healthy lean people, epicardial adipose tissue secretes cytoprotective and anti-inflammatory adipokines. However, in states of visceral adiposity, the epicardial adipose depot expands, especially in patients with HFpEF, particularly those who have worse LA and right ventricular function and PH.^26–28^ Simultaneously, the epicardial adipose secretory profile becomes proinflammatory, releasing adipokines that promote cardiac dysfunction and fibrosis. In 2012, Greulich et al^29^ described increased epicardial adipose tissue secretion of activin A in patients with metabolic disease, showing that it caused contractile dysfunction and downregulation of sarcoplasmic/endoplasmic reticulum calcium ATPase-2a in adjoining cardiomyocytes. Sarcoplasmic/endoplasmic reticulum calcium ATPase-2a suppression has been implicated in the pathogenesis of cardiomyocyte stiffening, particularly in obesity.^30^ Activin A was the first adipokine that was shown to be secreted from epicardial adipose tissue that had the potential to produce deleterious paracrine or vasocrine effects on the adjoining myocardium. Epicardial secretion of activin A is capable of producing fibrosis in the underlying atrial tissues.^31^

## ACTRII Signaling Modulates the Deleterious Response of the Heart and Pulmonary Vasculature To Injury and Stress

The transforming growth factor-beta (TGF-β) superfamily includes families of mutually reinforcing and antagonistic polypeptides (including activins, bone morphogenetic proteins [BMPs], and growth differentiation factors [GDFs]), which signal through activin type I and type II receptors (on the one hand) or through BMP type I and type II receptors (on the other hand), with the actions of the former being biologically oppositional to the actions of the latter.^32^ An imbalance that heightens ActRII signaling is proposed to drive the pathogenesis of many cardiac and vascular diseases.^4^

Among the known ActRII ligands, activin A is particularly well-characterized with respect to its cardiovascular actions. Other ligands include GDF-8 (which inhibits skeletal muscle mass), GDF-11 (which inhibits erythroid progenitor maturation), and BMP9 (which promotes vascular quiescence).^32^ Trapping of GDF-11 underlies the efficacy of activin type II ligand traps in treating anemia;^33^ and mutational loss-of-function of BMP9 has been linked to the development of telangiectasias.^34^

### Consequences of Activin A Signaling on the Left Atrium and Pulmonary Veins

Activin A binds directly to both subtypes of type II receptor (ActRIIA or ActRIIB), and the resulting activin type II complex recruits the ligand-specific type I receptor, i.e., activin-like kinase 4 (ALK4). The activin A-ActRII-ALK4 complex stimulates Smad2/3 signaling in the heart, which plays a central role in atrial and ventricular fibrosis (Figure 2).^35,36^ Activin A also drives cardiomyocyte senescence, apoptosis and stiffness and augments cardiac susceptibility to aging.^37^

**Figure 2.**
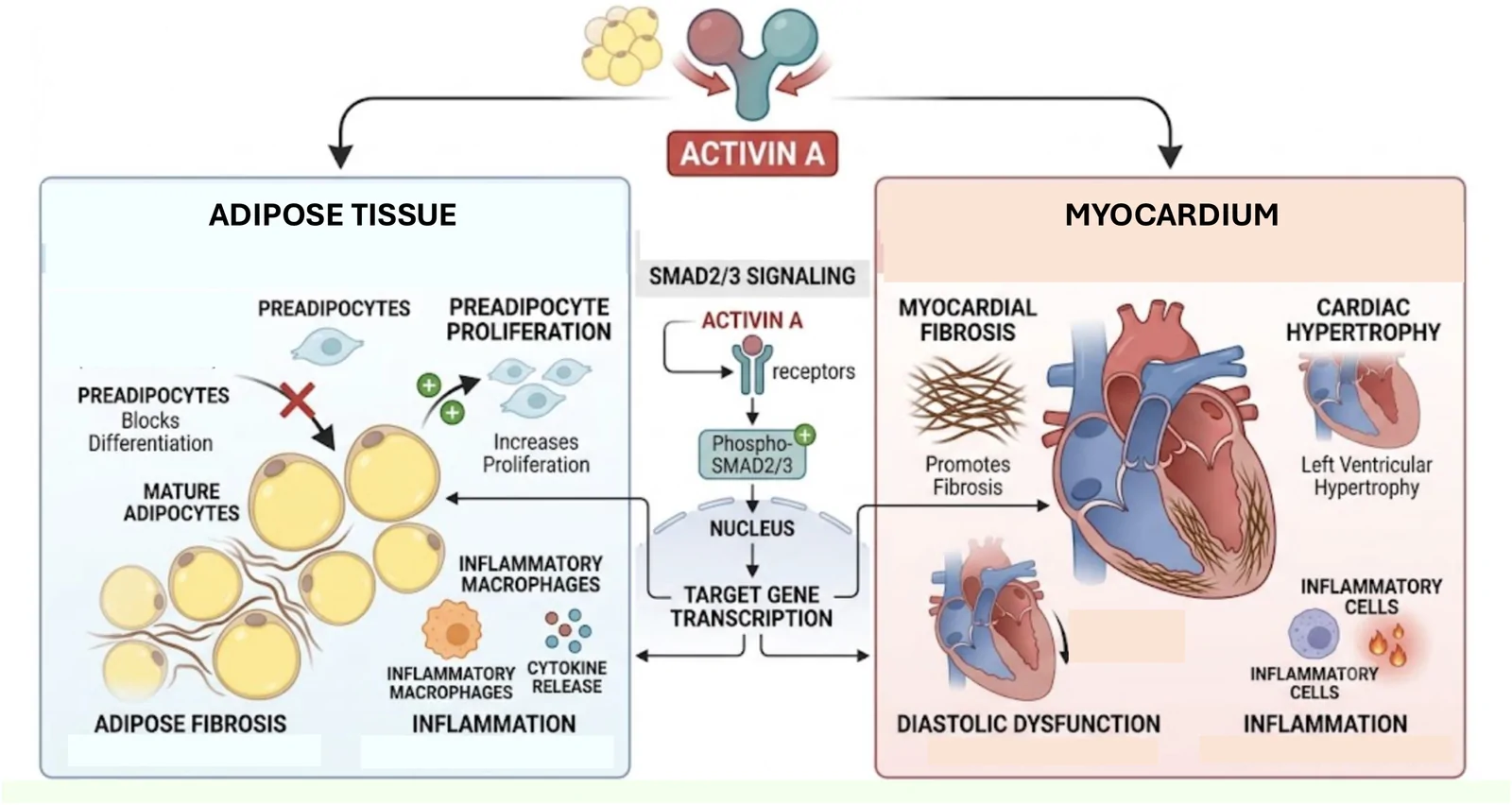
**Biological effects of activin A signaling on adipose tissue and the heart.** The secretion of activin A by an expanded adipose tissue mass acts to promote further hypertrophic expansion, inflammation, and fibrosis of fat depots, especially those surrounding the heart (ie, epicardial adipose tissue). Activin A promotes the proliferation of developing adipocytes (ie, preadipocytes) while attenuating their differentiation. Additionally, activin A promotes hypertrophy, fibrosis, and inflammation in the underlying myocardium and enhances stiffening of cardiomyocytes, thus promoting left atrial remodeling and dysfunction and impairing diastolic filling dynamics.

Elevated circulating levels of activin A are strongly associated with HFpEF. Patients with higher levels have greater LV mass and diastolic filling abnormalities, larger LA cavity sizes and higher right ventricular systolic pressures.^38^ Patients with left heart disease, pulmonary venous hypertension and increased PVR have higher circulating activin A levels than patients with pulmonary venous hypertension without increased PVR.^39^ Increased activin A expression and Smad2/3 signaling in the pulmonary vasculature lead to vascular endothelial and smooth muscle proliferation, fibrosis and adverse vascular remodeling in PH, and are implicated in the pathogenesis of pulmonary veno-occlusive disease.^40^ TGF-β1 (which signals through the ActRII) is an upstream regulator of the response of the PVs to mechanical stretch, and it is highly expressed in PVs in pulmonary venous hypertension due to left heart disease.^41,42^ In contrast, activin A does not play a major role in the remodeling of pulmonary arterioles in response to increased left heart pressures.^39^ Therefore, activin A secreted from epicardial adipose tissue and exerting paracrine and vasocrine effects on the underlying PVs and left atrium (but not on the PA) is well-positioned to play a role in the pathogenesis of CpcPH-HFpEF.

The proliferative and fibrotic actions of ActRII signaling are opposed by TGF-β ligands that signal through BMP type I and type II receptors, which mediate counter-regulatory antiproliferative and antifibrotic effects that are transduced through Smad1/5/9 transcription factors.^4^ Loss-of-function mutations in BMP type I or type II receptors are observed in patients with heritable and sporadic PAH,^43,44^ and BMP receptor and Smad1/5/9 signaling is attenuated both in experimental PAH and in experimental HFpEF induced by pressure overload or obesity.^45,46^

## The Adipokine Hypothesis of HFPEF, Activin a Signaling, and the Development of ACTRII Ligand Traps and Antagonists

What is the source of elevated activin A signaling in patients with HFpEF, pulmonary venous hypertension, and increased PVR? Activin A is synthesized in all cell types during stress, and cardiomyocytes can synthesize activin A in experimental myocardial infarction,^47^ but they are not reported to be a source of activin A in patients with HFpEF. In response to mechanical stretch, PV smooth muscle cells demonstrate enhanced TGF-β1 expression,^42^ but there is no evidence that intracavity hypertension drives increased activin A expression by LA or PV tissues. Importantly, in CpcPH, activin A is not involved in PA remodeling, and the PA is not known to be an important source of circulating ActRII ligands.^39,48^

Could the heart in HFpEF be influenced by activin A derived from an expansion of visceral adipose tissue mass? The adipokine hypothesis of HFpEF posits that hypertrophic expansion and proinflammatory transformation of visceral adipose tissue causes a change in its secretory profile of signaling molecules (eg, adipokines), which can exert paracrine, vasocrine, or endocrine effects to cause cardiomyocyte dysfunction, as well as cardiac inflammation, hypertrophy, and fibrosis, leading to HFpEF.^49^ Visceral adiposity precedes and predicts the development of HFpEF.^50^ It has been linked to HFpEF by Mendelian randomization studies,^51^ and it is present in the majority of patients with established HFpEF.^52^ Both visceral adiposity and HFpEF are characterized by suppression of adipose tissue secretion and circulating levels of cytoprotective and anti-inflammatory adipokines, and simultaneously, by an increase in adipose tissue secretion and circulating levels of pathogenetic adipokines that promote the structural and functional abnormalities of HFpEF.^53–55^ The epicardial fat depot is a particularly meaningful source of pathogenic adipokines, given its proximity to the heart and pulmonary vessels, and epicardial adiposity is correlated with LV concentric hypertrophy and the subsequent risk of heart failure.^56^ Excision of epicardial adipose tissue improves cardiac function after myocardial injury.^57^ A causal role for proinflammatory adipokines has been supported by studies of adipose-specific silencing of individual adipokines, which demonstrated that adipose tissue expansion and its secretion of pathogenic adipokines can cause HFpEF.^49,58–60^

### Adiposity and Increased Activin A Signaling

In its delineation of actionable pathogenic adipokines, the adipokine hypothesis identified activin A and ActRII signaling as key adipokine mediators in the pathogenesis of HFpEF.^49^ There exists a close relationship between adiposity, heightened ActRII signaling, and HFpEF. When visceral fat mass expands, the resulting local tissue hypoxia promotes signaling through hypoxia-inducible factor-1α, which then leads to increased expression of both activin A and ALK4 in human adipose progenitor cells.^61^ These changes promote adipose tissue hypertrophic expansion, inflammation, and fibrosis, and adipose-resident macrophages further induce activin A production (Figure 2).^62,63^ As a result of these mutually reinforcing mechanisms, visceral fat becomes a major source of systemic activin A synthesis in obesity, particularly because adipose tissue represents the largest endocrine organ of the body, representing 40% to 50% of total body weight in patients with obesity. These relationships explain why circulating levels of activin A are correlated with body mass index and epicardial fat thickness, and why bariatric surgery is accompanied by ≈30% to 40% decreases in circulating activin A levels.^64,65^

The production of activin A by visceral adipose tissue likely explains why circulating levels of activin A are increased in patients with HFpEF,^38^ particularly those with obesity, in proportion to the severity of LV hypertrophy and diastolic filling abnormalities, and have prognostic significance.^37,66^ Circulating levels of activin A can augment the intensity of cardiac ActRII signaling that is produced by activin A that is secreted from an expanded epicardial fat mass.^29,31,37^ Experimentally, activation of ALK4 (the type I receptor for activin A) promotes both atrial myopathy and HFpEF,^35,36^ and accordingly, activin type II ligand traps have been shown to alleviate experimental cardiac fibrosis and HFpEF induced by high-fat diet and metabolic diseases.^67,68^ A sotatercept analog ameliorates both the pulmonary vascular and left heart abnormalities in animals in which the phenotype of CpcPH-HFpEF is induced by obesity.^68^

### Suppression of Epicardial Adipose-Derived Adipokine Inhibitors of TGF-β Pathway Signaling in PH and HFpEF

Consistent with tenets of the adipokine hypothesis, the secretion of anti-inflammatory and anti-fibrotic adipokines (adiponectin, C1q/TNF [tumor necrosis factor]-related protein-9, omentin-1, and secreted Frizzled-related protein 5) is suppressed by obesity,^49^ and this suppression is also particularly noteworthy in epicardial adipose tissue harvested from patients with cardiovascular disease.^69–71^ These anti-inflammatory and anti-fibrotic adipokines antagonize the development of PH and HFpEF in experimental models and are deficient in pulmonary vascular disease.^49,72,73^ Adiponectin and C1q/TNF-related protein-9 inhibit TGF-β Smad2/3 signaling and fibrotic pathways in the heart,^74,75^ and omentin-1 acts as a direct natural decoy of ActRII ligands.^76^ In humans, omentin-1 is a signature adipokine secreted from epicardial adipose tissue, and its suppression is associated with atrial fibrosis;^77^ experimentally, it antagonizes pulmonary vascular remodeling and LV hypertrophy.^73,78^ Hence, PH and HFpEF are not only accompanied by increased activin A signaling, but they are also linked to suppression of anti-inflammatory and anti-fibrotic adipokines that act (at least in part) by inhibiting signaling through TGF-β pathways (Figure 3).^79^

**Figure 3.**
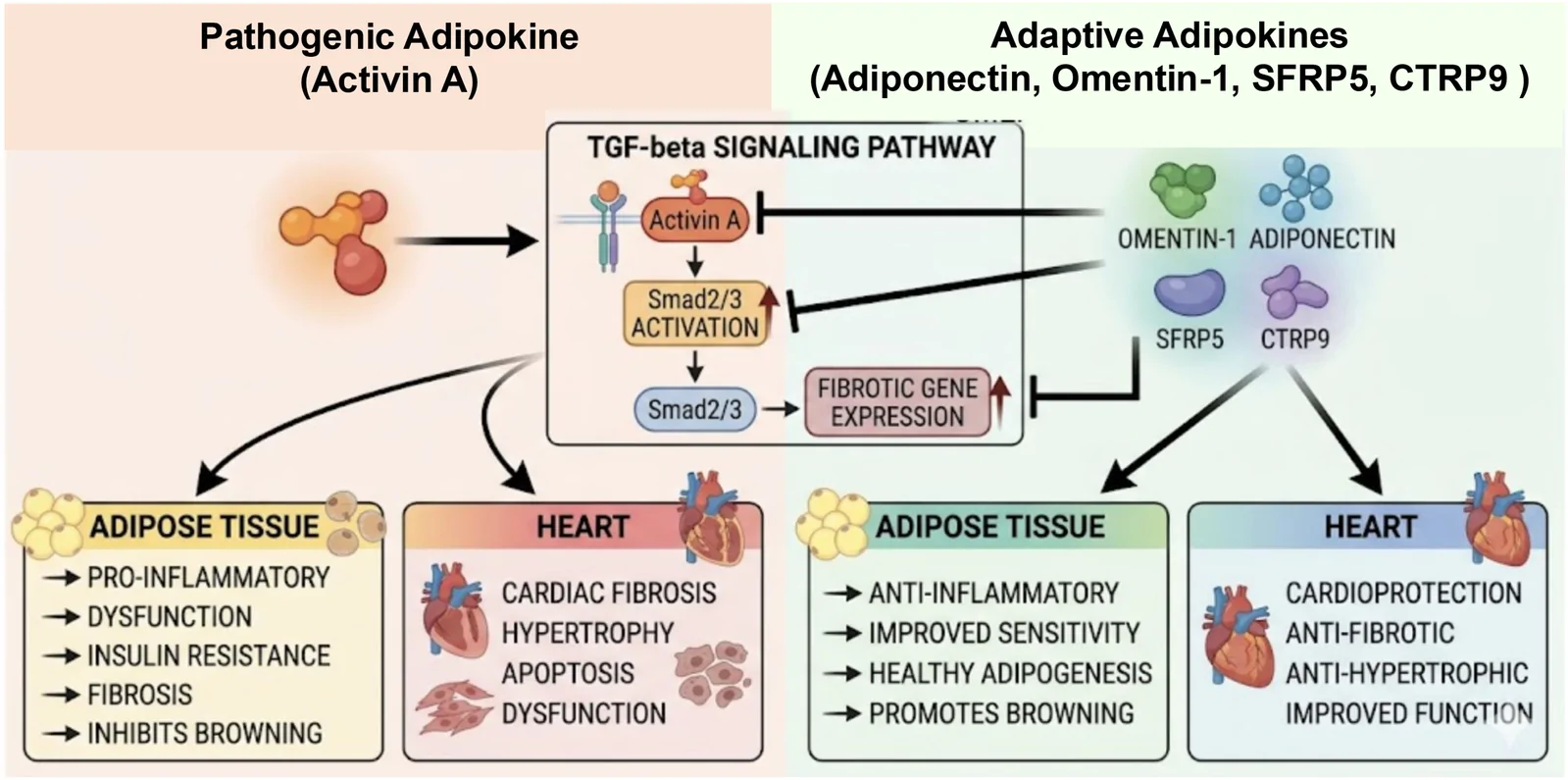
**Opposing effects of adaptive and pathogenic adipokines on adipose tissue and the heart.** The adipokine hypothesis posits that the evolution and progression of heart failure and preserved ejection fraction is driven by enhanced actions of pathogenic adipokines (eg, activin A) on the heart and adipose tissue, simultaneously with the suppression of the actions of adaptive adipokines (omentin 1, adiponectin, SFRP5 [secreted Frizzled-related protein 5], and CTRP9 [C1q/tumor necrosis factor (TNF)–related protein-9]). Both groups of adipokines are secreted from epicardial adipose tissue. The pathogenic adipokines promote adipose tissue hypertrophic expansion and inflammation, as well as cardiac hypertrophy, fibrosis, and dysfunction, effects that are opposed by the adaptive adipokines. Interestingly, the focal point of this critically important interaction involves members of the transforming growth factor-beta (TGF-β) superfamily, particularly activin A, acting as ligand that stimulates signaling through the activin type II receptor, leading to downstream signal transduction through Smad2/3.

### Pharmacological Approaches to Inhibition of ActRII Signaling

Diverse pharmacological approaches to activin A inhibition have been developed. An activin type IIA receptor ligand trap (eg, sotatercept) is formed by the fusion of the extracellular domain of the type IIA receptor to an immunoglobulin G–Fc segment, thus acting to sequester ligands in the extracellular space before they can engage cell-surface receptors. Sotatercept preferentially traps activin A, activin B, and GDF-11 (and, to a lesser extent, GDF-8);^1^ the sequestration of GDF-11 explains the effects of sotatercept to enhance erythropoiesis and increased red blood cell mass.^32–34^ It is possible that higher doses of sotatercept might trap receptor ligands that have adaptive cardiovascular effects, and thus, lower doses may act more selectively on activin A.^80^

Other approaches to activin A antagonism have been developed. Bimagrumab is an ActRII antagonist that binds to both type IIA and type IIB receptors, whereas sotatercept selectively traps type IIA receptor ligands. Bimagrumab is more likely to inhibit the myostatic action of GDF-8 (thereby promoting skeletal muscle mass), and it prevents ligand stimulation of ALK7 (thereby suppressing adiposity).^81^ Signaling through ALK7 can drive myocardial fibrosis in diabetic cardiomyopathy.^82^ Bimagrumab reduces pulmonary congestion and subclinical LV systolic dysfunction in experimental heart failure produced by aging or pressure overload.^37^ Another novel approach is the creation of a highly selective heterodimeric ligand trap (eg, ActRIIB:ALK4-Fc), which can selectively antagonize ActRII signaling occurring through ALK4, the type I receptor for activin A and GDF-11.^83^ ActRIIB:ALK4-Fc ameliorates the development of experimental HFpEF associated with diabetes.^84^ Drugs that bind extracellular activin A with high selectively (eg, garetosmab) have also been developed, and this approach may spare the binding of GDF-8, GDF-11, and BMPs.

## The Cadence Trial With Sotatercept in CPCPH-HFPEF: Design, Prospective Assessments and Key Findings

After the success of sotartercept in patients with PAH,^5,6^ the CADENCE trial evaluated the effects of the drug in a phase II trial in CpcPH-HFpEF.^85^ The trial randomized 164 patients who were assigned to placebo: 0.3 mg/kg every 3 weeks, and 0.7 mg/kg every 3 weeks, each for 24 weeks. The 0.7-mg/kg dose is the target dose of the drug for the treatment of PAH. The trial was envisioned as a proof-of-concept study for the effect of sotatercept on the pulmonary vasculature in CpcPH, and the placebo-corrected change in PVR was the primary end point. The measurement of circulating levels of activin A was not an entry criterion for participation in the trial.

### The Adipokine Hypothesis Anticipated an Effect of Sotatercept on the Left Heart in CpcPH-HFpEF

While the trial was ongoing, the adipokine hypothesis identified ActRII signaling as an important adipokine-mediated pathway for the development of HFpEF.^50^ Activin A acted as a key intermediary of the adverse effects of epicardial adipose tissue expansion on the adjoining myocardium.^29,31^ Circulating activin A levels were increased in HFpEF,^38^ and sotatercept analogs ameliorated left-heart abnormalities in experimental HFpEF.^68^ Accordingly, it was envisioned that sotatercept might target a pathogenic adipokine in HFpEF, leading to the hypothesis that phase II trials with sotatercept would show favorable effects on the left heart in patients with HFpEF.^49^ Changes in PAWP and LA volume index were assessed at baseline and after 24 weeks.

### Principal Findings of the CADENCE Trial

The CADENCE trial enrolled primarily elderly women with obesity and HFpEF (75 years of age; body mass index, 31 kg/m^2^, with PVR, 5.2 Wood units). Half the patients were receiving sodium-glucose cotransporter 2 inhibitors or mineralocorticoid receptor antagonists. The baseline clinical and hemodynamic features were consistent with patients with HFpEF enrolled in large-scale clinical trials.

After 24 weeks of double-blind treatment, for the primary end point, sotatercept reduced PVR with both the 0.3-mg/kg and 0.7 mg/kg-dose groups (by 1.0 and 0.75 Wood units, respectively); however, the magnitude of these effects are one-third of those reported with sotatercept in PAH.^5,6^ Mean PA pressures declined on average by 9.1 mm Hg in each treatment group, and N-terminal prohormone B-type natriuretic peptide levels decreased by 344 and 402 pg/mL (in the 0.3-mg/kg and 0.7 mg/kg-dose groups, respectively); these changes were smaller than those observed in PAH trials. Functional capacity improved with both doses, although more with the 0.3-mg/kg dose.

Importantly, 0.3 mg/kg of sotatercept decreased PAWP by 3.0 mm Hg and decreased LA volume index by 6.4 mL/m^2^ (both *P*<0.05). These changes were larger than those reported for drugs currently recommended for use in HFpEF.^86–90^ The changes in PAWP and LA volume index in the 0.3-mg/kg group were somewhat larger than with 0.7 mg/kg, paralleling the dose-response relationships seen for PVR. The observed decreases in PAWP and LA volume index with sotatercept are noteworthy, especially because they have not been observed with conventional pulmonary vasodilators. Of particular note, sotatercept significantly increased systemic vascular resistance and raised systolic blood pressure (by 4.5 mm Hg), actions that would have been expected to increase, not decrease, left heart pressures and volumes.

A worsening clinical event was defined as the composite of all-cause death, nonelective hospitalization ≥24 hours for PH or heart failure, use of parenteral diuretics, or ≥15% decrease from baseline in 6-minute walk distance in 2 tests. Risk estimates were lower in the 0.3 mg/kg group (hazard ratio, 0.16; 95% CI, 0.05–0.62) and in the 0.7 mg/kg group (hazard ratio, 0.59; 95% CI, 0.25–1.36), with nominal significance only for the 0.3 mg/kg group. Sotatercept increased hemoglobin by 1.6–1.8 g/dL.

It should be noted that these observations derive from a phase II proof-of-concept trial that was not designed to examine the effect of sotatercept on worsening heart failure events. As a result, the number of events were few, yielding wide CIs. The duration of treatment was limited to 24 weeks, and patients with HFpEF with a PVR <4 units were not enrolled, although they represent an appropriate group for further study. These limitations are being addressed in a formal phase III outcomes trial.

## Summary and Conclusions

The totality of available evidence suggests that the mechanisms underlying the increase in PVR in patients with CpcPH-HFpEF may differ meaningfully from those that are responsible for the increase in PVR in PAH. Pulmonary arterial vasodilators that lower PVR and improve clinical status in PAH do not also produce hemodynamic or symptomatic benefits in patients with CpcPH-HFpEF. These disappointing results may be best explained if CpcPH-HFpEF is envisioned as a disorder that primarily afflicts the PVs and LA main body. Epicardial adipose tissue lies in intimate anatomical proximity to both structures, allowing its secretory products to influence their structure and function.

The adipokine hypothesis of HFpEF posits that the expansion and biological transformation of visceral fat depots (especially epicardial adipose tissue) lead to the secretion of adipokines, which cause the pathophysiological derangements in the heart, kidney, and vasculature that are highly characteristic of patients with HFpEF. Elevated circulating levels of the adipokine activin A are strongly associated with HFpEF, particularly patients with larger LA cavity size, pulmonary venous hypertension, and increased PVR. Signaling through the ActRII regulates the response of the PVs to mechanical stretch, being augmented in the PVs in states of pulmonary venous hypertension due to left heart disease.

Activin A signaling promotes the development of both atrial myopathy and HFpEF in experimental models, and ActRII antagonism alleviates experimental HFpEF induced by high-fat diet and metabolic diseases. ActRII ligand traps ameliorate both the pulmonary vascular and left heart abnormalities in animal models of obesity-induced CpcPH-HFpEF, leading to the hypothesis that sotatercept would produce favorable effects on the left heart in patients with CpcPH-HFpEF. These expectations were supported by the CADENCE trial, which demonstrated that sotatercept reduced both PAWP and LA volume index in patients with CpcPH-HFpEF, effects that could not be attributed to the concomitant decrease in PVR. The ability of sotatercept to slow the progression of CpcPH-HFpEF will be tested in a phase III outcomes trial.

## Article Information

### Disclosures

Dr Packer reports consulting fees from 89bio, Abbvie, Actavis, Altimmune, Alnylam, Amgen, Ardelyx, ARMGO, AstraZeneca, Attralus, Biopeutics, Boehringer Ingelheim, Caladrius, Casana, CSL Behring, Cytokinetics, Daiichi Sankyo, Imara, Lilly, Medtronic, Merck Moderna, Novartis, NovoNordisk, Pharmacocosmos, Regeneron, Roche, Salamandra, and v-Wave. Dr Gomberg-Maitland has served as a consultant for Acceleron Pharma, Inc, Merck & Co, Inc, Rahway, NJ, Allorock/Jucabio, Janssen Biotech, Inc, Keros Therapeutics, and United Therapeutics Corp; and reports that her spouse/partner is an employee of Pathos AI, Inc. Dr Tedford serves on steering committees for Abbott, Edwards, Endotronix, Gradient, Merck, Restore Medical, and Tempus AI, as well as a research advisory board for Abiomed. Dr Langleben reports a relationship with Merck and Co Inc. that includes: consulting or advisory, funding grants, speaking and lecture fees, and travel reimbursement; a relationship with Actelion, a business unit of Janssen-Cilag Pty Ltd. that includes consulting or advisory and speaking and lecture fees; a relationsip with Bayer Corporation that includes consulting or advisory; a relationship with Novartis Pharmaceuticals that includes consulting or advisory; and a relationship with PhaseBio Pharmaceuticals Inc that includes consulting or advisory. Dr Vachiery reports consulting fees from Merck, BPI France, Gossamer Bio, Liquidia, Pulmovant, Tectonic Therapeutics, Inhibikase Therapeutics, and Insmed, speaker fees from Janssen, Boehringer, and Novartis, support for meeting attendance from MSD and patents with GK and J&J. Dr Borlaug has served as a consultant for Actelion, Amgen, Aria, Axon Therapies, BD, Boehringer Ingelheim, Cytokinetics, Edwards Lifesciences, Lilly, Imbria, Janssen, Merck, Novo Nordisk, NGM, NXT, and VADovations, and is named inventor (US patent No. 10,307,179) for the tools and approach for a minimally invasive pericardial modification procedure to treat heart failure.
